# Correlation of left atrial wall thickness and atrial remodeling in atrial fibrillation: Study based on low-dose-ibutilide-facilitated catheter ablation

**DOI:** 10.1097/MD.0000000000015170

**Published:** 2019-04-12

**Authors:** Kun Zuo, Kuibao Li, Min Liu, Jing Li, Xiaoqing Liu, Xingpeng Liu, Jiuchang Zhong, Xinchun Yang

**Affiliations:** a Heart Center & Beijing Key Laboratory of Hypertension; b Department of Radiology, Beijing Chaoyang Hospital, Capital Medical University, Beijing, China.

**Keywords:** atrial fibrillation, atrial remodeling, ibutilide-facilitated catheter ablation, LA wall thickness, substrate

## Abstract

Atrial remodeling plays a significant role during the progression of atrial fibrillation (AF). Left atrial wall thickness (LAT) is a subjective and easily acquirable indicator referring to structural remodeling. Therefore, we aimed to investigate the association between LAT and atrial remodeling substrate, and to explore the predictive role of LAT about strong maintenance substrate and poor response to catheter ablation.

LAT was measured by cardiac computed tomography in 2 selected locations (roof and floor) in 100 persistent AF patients. Then the low-dose-ibutilide-facilitated catheter ablation was performed and atrial maintenance substrate was categorized as weak, mild, and strong, based on the response to circumferential pulmonary vein isolation or complex fractionated atrial electrograms ablation. During follow-up, the success rate was evaluated. LAT showed a progressive thickening tendency from weak, mild, to strong maintenance substrate (roof: 2.2 mm vs. 2.6 mm vs. 3.9 mm, *P* < .0001; floor: 1.7 mm vs. 2.0 mm vs. 2.5 mm, *P* < .0001). During follow-up, the success rate of ablation was decreased with the maintenance substrate strengthening (weak 80%, mild 64.53%, strong 31.43%, *P* = .009). LA roof thickness >3.10 mm might be the predictor to strong atrial maintenance substrate and poor response to ablation.

LAT was associated with the remodeling extent of atrial maintenance substrate and might predict the response to catheter ablation. These findings could help the clinicians to select the appropriate ablative strategy and predict the complexity and prognosis before catheter ablation.

## 1 Introduction

Atrial fibrillation (AF) is the most common arrhythmia with worldwide prevalence, increased disability, and morbidity.^[1–3]^ Although catheter ablation has been successfully used for alleviating the symptom of palpitation,^[4]^ AF continues to perplex researchers with its incompletely understood mechanisms and poor response to ablation in non-paroxysmal AF patients. The heterogeneity of underlying atrial substrate, extent of atrial fibrosis, and the discrepancies between interindividual electrophysiological characteristics contribute to unpredictable responses to drug or ablation therapy.^[5]^ Atrial remodeling or atrial maintenance substrate plays a significant role during AF progression.^[6]^ Therefore, adequate assessment about atrial remodeling is crucial to select an appropriate therapeutic strategy. Low-voltage area, complex fractionated atrial electrograms (CFAEs) and rotors are some of the popular targets observed in the current mapping process, but are often affected by objective and subjective factors, such as the mapping density. Meanwhile, some popular indicators, such as atrial fibrosis ^[7]^ and left atrial wall thickness (LAT), could reflect the remodeling substrate subjectively. However, the quantification of atrial fibrosis was impeded mostly by the longer examination period of delayed enhancement magnetic resonance imaging and non-widespread analyzing software. Therefore, the present study was conducted to explore the potential association between LAT and atrial maintenance substrate, based on the response to low-dose-ibutilide-facilitated catheter ablation. In addition, we evaluated whether a thickened left atrial wall was associated with a poor response to catheter ablation.

## 2 Methods

### 2.1 Study population

This prospective study included 100 patients who underwent a first-time ablation procedure for symptomatic, persistent nonvalvular AF (54 men, 46 women; mean age 63.03 ± 9.78 years) in Beijing Chao-Yang Hospital. Persistent AF was defined as a sustained episode lasting >7 days.^[8]^ Exclusion criteria included: New York Heart Association (NYHA) grading III or IV; severe hypertrophic cardiomyopathy (>20 mm); baseline QTc >500 ms; left atrial appendage thrombosis.^[9]^ Demographic and clinical characteristics were obtained by completing face-to-face surveys and checking hospital or medical examination records. The study was conducted in accordance with the principles specified for research on human subjects in the Declaration of Helsinki and the research protocol was approved by the ethics committee of Beijing Chaoyang Hospital. All of the participants signed informed consents.

### 2.2 Cardiac CT imaging

Contrast-enhanced cardiac computed tomography (CT) imaging was performed with a dual-source CT scanner (SOMATOM Definition; Siemens Healthcare, Erlangen, Germany) within 3 days preceding the ablation. A mechanical injector was used to intravenously inject a bolus of 370 mg/mL iopromide (Ultravist; Bayer Healthcare Pharmaceuticals, Berlin, Germany) at a flow rate of 5.0 mL/s followed by a 50 mL saline flush. Parameters for imaging were: detector collimation, 2 × 32 × 0.6 mm; slice acquisition, 2 × 64 × 0.6 mm by the means of a z-flying focal spot; and gantry rotation time, 330 ms.

### 2.3 CT-based measurement of LAT

**Figure 1:**
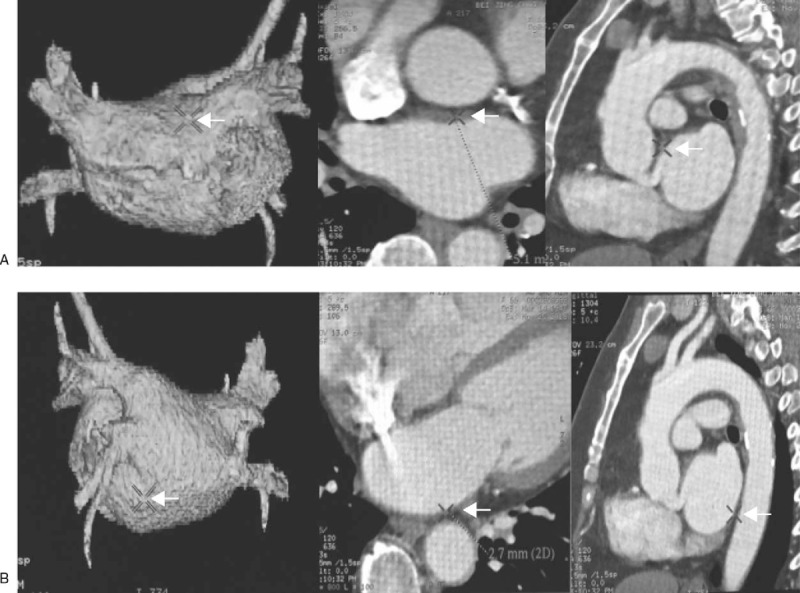
Measurement of the thickness of LA roof and floor. Left atrial wall thickness was measured at 2 preselected locations by using the axial plane image, roof (A) and floor (B). And measurements would be taken with electronic calipers at 3 sites, which were located within 5 mm of the thickest part of the LA, and the average was considered for further analysis. LA = left artery.

Acquired images were transferred to a workstation (ADW 4.2; GE Healthcare, Boston) where epicardial fat was detected by assigning Hounsfield units from −50 to −200 to fat, to be excluded from the LAT measurement. To validate the difference between the LA wall and epicardial fat, we measured and compared the Hounsfield units at 3 randomly selected sites on the LA wall and at 3 other sites with fat in each AF patient. Wall thickness was measured at 2 preselected locations, LA roof (Fig. 1A) and LA floor (Fig. 1B), using the axial plane image. At each location, measurements were taken with electronic calipers at 3 sites, which were located within 5 mm of the thickest part of the LA, and the average was considered for analysis.

### 2.4 Low-dose-ibutilide-facilitated catheter ablation and category of atrial maintenance substrate

After double transseptal punctures guided by a three-dimensional (3D) electroanatomic mapping system (CARTO 3, Biosense Webster, Irvin, CA), a 3D reconstruction of the LA was obtained using a circular mapping catheter (NaviStar Thermocool, Biosense Webster) and this was merged with the 3D VR cardiac CT. After circumferential pulmonary vein isolation (CPVI), low-dose (0.25 mg) ibutilide was infused intravenously over 3 minutes in patients in whom persistent AF could not be terminated.

The following ablation strategy was depended on the response of ibutilide.^[10]^ In cases where AF was terminated by ibutilide within 30 minutes, no further ablation would be taken. While in cases whose rhythm converted to atrial tachycardia (AT) within 30 minutes, the specific ablation would be taken until the sinus rhythm (SR) was turned. In contrast, in the patients with persisting AF, the 3D electroanatomic representation of the LA would be reconstructed and CFAEs would be mapped in the model. CFAEs were defined as the common criteria ^[11]^ and were mapped within LA and coronary sinus (CS). The destination of CFAEs’ ablation was either the restoration of SR or AT. And for AT, the specific ablation would be taken through the targeted mapping until the SR was turned. While the CFAEs of LA and CS had been eliminated or the CFAEs ablation time reached 30 minutes, electrical cardioversion was to be undertaken for SR restoration.

**Figure 2:**
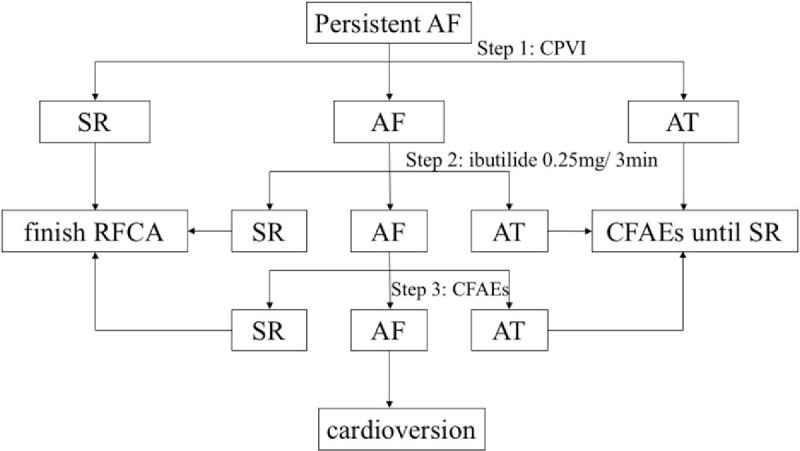
Flowchart of low-dose-ibutilide-facilitated RFCA. In cases where AF was terminated by ibutilide within 30 minutes, no further ablation would be taken. While in cases whose rhythm converted to atrial tachycardia (AT) within 30 minutes, the specific ablation would be taken until the sinus rhythm (SR) was turned. In patients with persisting AF, the 3D electroanatomic representation of the LA would be reconstructed and CFAEs would be mapped and ablated until the restoration of SR or AT. While the CFAEs of LA and CS had been eliminated or the CFAEs ablation time reached 30 minutes, electrical cardioversion was to be undertaken for SR restoration. AF = atrial fibrillation, CFAEs = complex fractionated atrial electrograms, LA = left artery.

The block of ablation lines was identified in SR or pacing rhythm. If the isolation were not continued, a corresponding ablation would be delivered. After CPVI, adenosine triphosphate was injected intravenously to provoke dormant pulmonary vein conduction. Radiofrequency energy was applied, at 30 to 35 W with discharging time of 30 second for each point, to the dormant PV conduction sites until PV conduction disappeared (as shown in Fig. 2).

Based on the response of low-dose-ibutilide-facilitated catheter ablation, the extent of atrial maintenance substrate was categorized as 3 group, weak (responsive to CPVI), mild (responsive to CPVI+CFAEs), and strong (unresponsive to CPVI+CFAEs).

### 2.5 Postoperative treatment and follow-up

Following catheter ablation, antiarrhythmic medications would be discontinued at the 3rd month, and warfarin at the 6th month, respectively, with INR maintained between 2 and 3. At 1, 3, 6, 9, 12, 18, and 24 months, patients underwent 72-hour Holter monitoring; an electrocardiogram was recorded if any patient complained of discomfort to detect AF recurrence or LA re-entry tachycardia after catheter ablation. Successful ablation was determined as the absence of episodes of AF or AT (>30 seconds) without antiarrhythmic treatment after the blanking period (3 months).

### 2.6 Statistical analysis

Normal continuous variables were presented as mean ± standard deviation. Continuous data were analyzed by 1-way ANOVA to test for significant differences. Abnormal continuous variables were shown as median and were analyzed by the Kruskal–Wallis test. Differences in categorical variables were analyzed by Fisher's exact test. Multivariate analysis was conducted with the ordered logistic regression model reporting coefficient (Coef.). AF recurrence was analyzed in a Kaplan–Meier survival analysis. The area under the receiver operating characteristic (ROC) curve was used to evaluate the prognostic value of the LAT. Differences were considered statistically significant at values of *P* < .05. Statistical analysis was performed with the SPSS v.22.0 statistical package (SPSS Inc., Chicago, IL), STATA 14 (Stata Corp, College Station, TX), and Microsoft Excel 2011 (Microsoft Corp, Redmond, WA).

## 3 Results

### 3.1 Clinical characteristics of study subjects

The clinical characteristics of all subjects are shown in Table 1. There was no significant difference between 3 groups in terms of age, sex category, body mass index, coronary heart disease, hypertension, diabetes mellitus, and stroke. Of note, the left atrial size, reflected by the left atrial diameter (LAD), left atrial length diameter (LALD), and left atrial transverse diameter (LATD) derived from echocardiogram, was significantly larger in the mild and strong group. Moreover, the decreased cardiac function reflected by left ventricular ejection fraction (LVEF) and N-terminal pro-brain natriuretic peptide (NT-proBNP) showed no specific correlation within the different maintenance substrate.

**Table 1:**
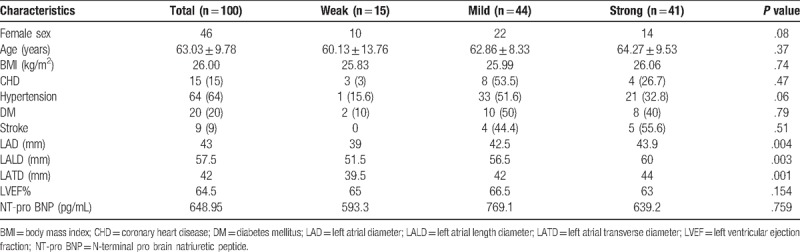
Baseline clinical characteristics of the study cohort.

### 3.2 Low-dose-ibutilide-facilitated catheter ablation and category of atrial maintenance substrate

**Figure 3:**
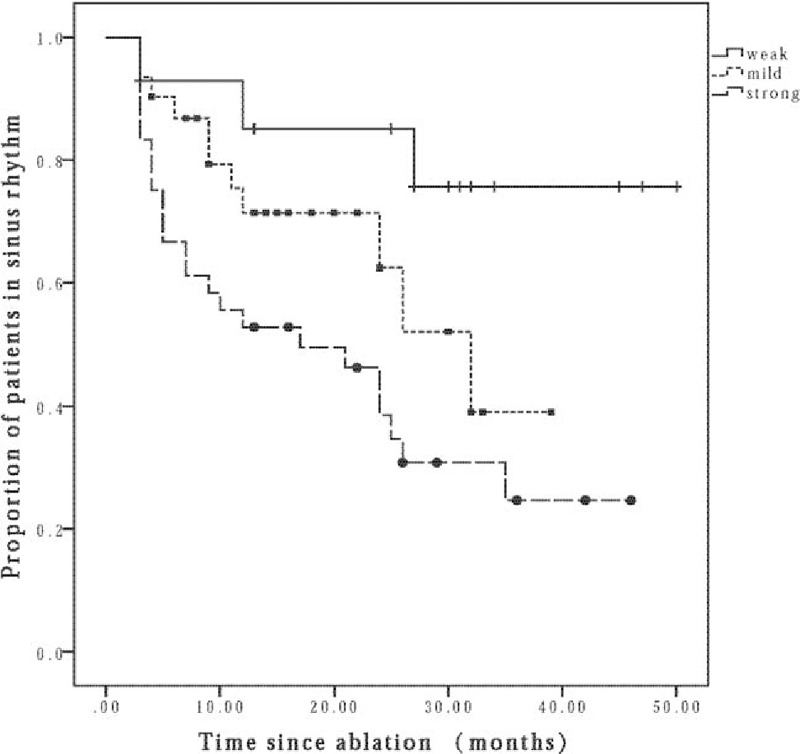
Kaplan–Meier survival analysis of patients in normal SR after ablation. The Kaplan–Meier survival analysis of patients in normal sinus rhythm after ablation. Significant differences in the AF-free survival rate were revealed among the 3 groups (weak 80%, mild 64.53%, strong 31.43%, *P* = .009). AF = atrial fibrillation.

In order to assess the extent of LA remodeling, we evaluated the atrial maintenance substrate based on the response to low-dose-ibutilide-facilitated catheter ablation and then categorized the atrial maintenance substrate as: weak (n = 15): responsive to CPVI, where persistent AF can be converted to SR or AT by CPVI or/and low-dose ibutilide. These included 14 patients converted to SR and 1 patient to AT after ibutilide injection. Mild (n = 44): CPVI and CFAEs ablation could restore the cardiac rhythm to SR (n = 23) and AT (n = 21). Strong (n = 41): unresponsive to CPVI+CFAEs, SR could be restored until electrical cardioversion performed. There was no complication in the electrophysiological examination and ablation. And the participants received antiarrhythmic therapy during the first 3 months after ablation, 79 of them took amiodarone, 7 of them took propafenone, and 3 of them received sotalol therapy. With a mean follow-up duration of 16.3months (range 3–50 months) after ablation, the success rate of ablation showed a declining trend with the maintenance substrate strengthening (weak 80%, mild 64.53%, strong 31.43%, *P* = .009). Furthermore, the recurrence rate among AF patients who received different types of antiarrhythmic therapy was similar (*P* = .186, amiodarone vs. propafenone; *P* = .367, amiodarone vs. sotalol; *P* = .266, propafenone vs. sotalol). Figure 3 showed the results of the Kaplan–Meier survival analysis of patients in normal SR after ablation. Significant differences in the AF-free survival rate were revealed among the 3 groups.

### 3.3 LAT in different atrial maintenance substrate groups

As revealed by echocardiology, LA significantly enlarged with the LA maintenance substrate strengthening, and this seemed to be in accordance with our previous acknowledgment that LA enlargement is a traditional risk factor for AF. However, a complex remodeling process which may refer to inflammation^[12]^ was involved in the duration from normal to enlarged left atrium. Therefore, we measured the LA wall thickness and found the potential association between LAT and different category of LA maintenance substrate. Overall, the thickness of LA presented a thicker tendency with the stronger substrate (roof: 2.2 mm vs. 2.6 mm vs. 3.9 mm, respectively, *P* < .0001; floor: 1.7 mm vs. 2.0 mm vs. 2.5 mm, respectively; *P* < .0001).

Correlation between the category of atrial substrate and potential covariates is shown in Table 2. A moderately positive correlation was found between the category of substrate and the wall thickness of LA roof (*r* = 0.773, *P* < .0001), floor (*r* = 0.581, *P* < .0001). Wall thickness of LA roof and floor was significantly associated with the category of atrial substrate (roof: Coef. 3.97, *P* = .000; floor: Coef. 2.20, *P* = .002).

**Table 2:**
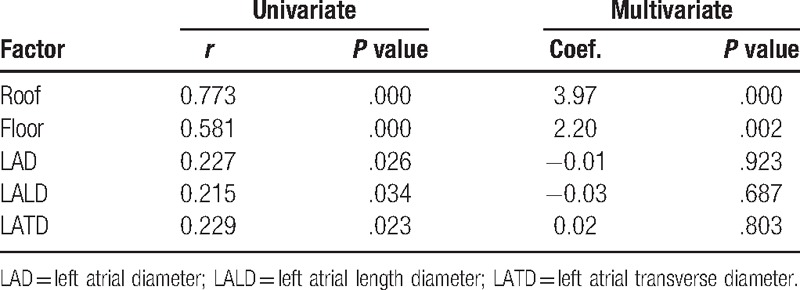
Potential covariates related to the category of atrial maintenance substrate.

### 3.4 LAT as a predictor of poor response to ablation

**Figure 4:**
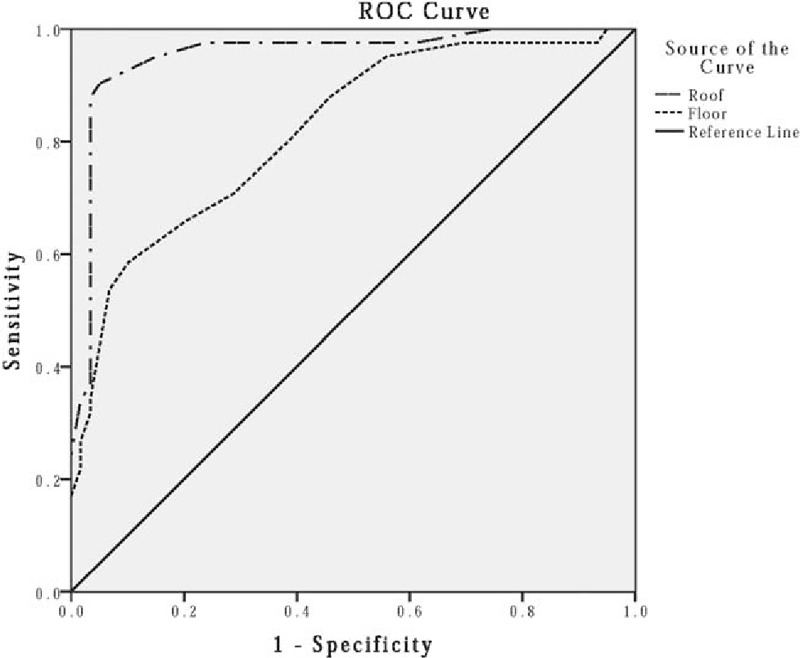
ROC curves of LAT for prediction of atrial substrate. The ROC curves of LAT for prediction of atrial substrate with an area under the curve (AUC) of LA roof: 0.954 (95% confidence interval [CI] 0.909–0.999, *P* < .0001) and LA floor: 0.820 (95% CI 0.737–0.903, *P* < .0001). ROC = receiver operating characteristic curve, LAT = left atrial wall thickness, LA = left artery.

Analysis of ROC curves (Fig. 4) with an area under the curve (AUC) of LA roof: 0.954 (95% confidence interval [CI] 0.909–0.999, *P* < .0001) and LA floor: 0.820 (95% CI 0.737–0.903, *P* < .0001) revealed that LAT of 3.10 mm (roof, sensitivity 90.2%, specificity 94.9%) and 2.35 mm (floor, sensitivity 58.5%, specificity 89.8%) were the optimal cutoff values predictive of strong atrial maintenance substrate, which might be the predictive indicator for poor prognosis of ablation. So far, we have identified the potential association between LAT and atrial remodeling substrate and explored the predictive value for LAT in detecting strong left atrial maintenance substrate and poor response to ablation in AF patients.

## 4 Discussion

In the present study, we obtained seminal evidence delineating the association between thickness of left atrial wall and remodeling extent of maintenance substrate based on cardiac CT and response to low-dose-ibutilide-facilitated catheter ablation in a cohort of AF patients. The thickness of the left atrial wall tended to be thicker, not a stretch-related thinning tendency, when the atrial maintenance substrate got stronger. A thickened left atrial wall was associated with a poor response to ablation. LAT may be considered as a relatively objective indicator to predict the remodeling extent of atrial maintenance substrate and prognosis to catheter ablation.

### 4.1 LAT and AF

LAT has been shown thickened in a stepwise fashion, ranging from control subjects to patients with paroxysmal AF to those with persistent AF.^[13]^ Other research has indicated that the LA wall was thicker at CFAEs sites.^[14]^ Our findings further explored the thickening trend in correlation with strengthening of the atrial maintenance substrate. A thickened left atrial wall may induce structural remodeling. Histologic study of LA tissue in chronic AF patients ^[15]^ and rapid atrial pacing goat model ^[16]^ revealed the severe loss of myocytes, glycogen accumulation, changes in mitochondrial morphology, and cellular hypertrophy. Taken together, the thickened left atrial wall is associated with structural remodeling, which may accelerate AF progression. Furthermore, Nakatani et al^[17]^ showed that thick left atrial wall contributes to AF recurrence after ablation, which was in accordance with the present study.

In addition, we selected roof and floor as representative of LA in the present study because they were supported by advantages of relatively accurate spatial orientation, unusual anatomic variants, and, particularly, common targets for AF substrate modification strategy. Other regions such as posterior, septal, or lateral wall of LA may associate with atrial remodeling either, but unsuitable to measure due to poor consistency or spatial orientation. Roof and floor could only represent part of LA, but it has been already identified with reasonable association in the present study.

Of note, the present study showed that thicker left atrial wall was associated with stronger atrial maintenance substrate, but with an enlarged left atrium measured by LAD from echocardiology. An enlarged LA and a thickened LA wall might seem to be contradicted. Actually, thickened left atrial wall may implicate the stage of inflammation, edema. With progression of AF to end-stage disease, remodeling may advance to atrial fibrosis, attendant to fractionated electrical activities with low voltage on the electroanatomic mapping that may be predictive of the high risk of AF initiation and persistence. Loss of adaptive atrial thickening may be the tipping point at which fibrosis and scar become irreversible and cardiac function is impaired.^[18]^ We excluded heart failure patients with NYHA Class III, IV due to their poor tolerance of catheter ablation; therefore, our results may only reflect the adaptive reserve state before cardiac function is irreversibly impaired.

### 4.2 Ibutilide-facilitated AF catheter ablation

CPVI, which can interrupt abnormal electrical activities between the PVs and the LA, is the cornerstone of catheter ablation in AF. But for non-paroxysmal AF, the result of simple CPVI is generally unsatisfactory because of the presence of the complex substrate. Additional PV triggers were found frequently while mapping. Therefore, various strategies for atrial maintenance substrate modification are often applied in AF ablation procedures. The “roof line” and the “mitral isthmus line” showed an additive effect when compared with CPVI alone.^[19]^ Another atrial maintenance substrate modification strategy is CFAEs ablation, which correlates with slowed conduction and pivot points of wavelets.^[20]^ As many CFAEs sites are nonspecific and reflect passive atrial activation not critical to AF maintenance, extensive ablation may also be unnecessary.^[21]^ A preclinical study identified that ibutilide, a class III antiarrhythmic drug, can limit atrial re-entry without impacting focal sources of AF in cases with persistent AF.^[22–24]^ Meanwhile, some studies have also shown that the use of ibutilide in the context of a stepwise ablation could result in reduction of fractionation and higher rates of AF termination.^[25]^ In our study, we performed the advantages of ibutilide to identify the sites that were critical for persistent AF maintenance to improve procedural efficacy and to categorize the left atrial maintenance substrate.

### 4.3 Atrial remodeling substrate in AF

Atrial remodeling, substrate plays a key role throughout the progression of AF. With the establishment of a novel therapeutic concept about fibrotic atrial cardiomyopathy (FACM),^[26]^ atrial fibrosis has been valued. Extensive atrial fibrosis is always associated with poor outcomes following ablation.^[5]^ Atrial fibrosis has been postulated as a potential cause of the abnormalities in the wave-front perpetuation and the formation of circuits necessary for re-entry, which may increase the underlying risk of initiation and maintenance of AF. Quantitative and qualitative assessments of FACM are often indicated by Late Gadolinium-Enhanced Cardiac MRI (LGE-CMRI). However, visualization and quantification of scar tissues have always been the difficulty during the clinical practice of LGE-CMRI. Recently, a novel fully automated multiview 2-task recursive attention mode has been designed, which can segment the LA and PV anatomy and the atrial scars directly, and generate a patient-specific anatomical and atrial scar assessment model.^[27]^ Therefore, the development of technology about assessment of the atrial maintenance substrate helps the electrophysiologist to stratify patients, guide ablation therapy, and predict treatment success.

From the present study, LAT is an easy-acquired indicator which associated with atrial remodeling substrate and should be taken into consideration to perform an appropriately patient-tailored ablation strategy and predict the complexity and prognosis of ablation. In the future, it can potentially be extended to a multicenter study to establish a risk score system for identification of the left atrial substrate in AF patient population.

### 4.4 Potential limitations

Consideration of possible limitations is of relevance to our study and help to inform the design of future studies. We semi-quantitatively assess the remodeling extent of AF substrate based on the response to ibutilide during catheter ablation as weak, mild, and strong. The correlation between LAT and some other quantitative indicators, such as electrophysiologic characteristics involving low-voltage area or CFAE index, could provide more precise and valuable guidance in clinical practice. Furthermore, the histologic characteristics of LAT remain uncertain. Measurement of LAT, interstitial fibrosis, and inflammation in AF samples and analyzing their correlation with remodeling extent could provide stronger evidence. The present results provided preliminary clues and evidence for future investigations regarding the potential mechanisms between LAT and atrial remodeling in AF patients and further studies are still needed.

## 5 Conclusions

The present study provides the first description about the association between thickness of left atrial wall and remodeling extent of maintenance substrate in a cohort of AF patients. These novel findings are fundamental for further studies connecting an easily obtained indicator from cardiac CT with remodeling substrate while electrophysiologic mapping, but they are just the beginning. An extensive amount of research is still needed to explore the clinical values of LAT to reflect the remodeling extent of substrate precisely.

## Author contributions

**Conceptualization:** Kun Zuo, Xinchun Yang.

**Data curation:** Kun Zuo, Kuibao Li.

**Formal analysis:** Kun Zuo, Kuibao Li, Jing Li.

**Investigation:** Kun Zuo.

**Methodology:** Kun Zuo, Kuibao Li, Min Liu.

**Project administration:** Xinchun Yang.

**Software:** Kuibao Li.

**Supervision:** Xinchun Yang.

**Writing – original draft:** Kun Zuo.

**Writing – review & editing:** Kuibao Li, Xiaoqing Liu, Xingpeng Liu, Jiuchang Zhong, Xinchun Yang.
